# Delayed diagnosis and management of necrotizing fasciitis of the left lower leg: A case report

**DOI:** 10.1097/MD.0000000000031231

**Published:** 2022-10-28

**Authors:** Li-Xia Zhang, Zhao-Jun Liang, Bao-Yin Zhao, Xue-Wen Shi, Tao Zhang, Hua Liu, Xiao-Hui Yu

**Affiliations:** a The 940^th^ Hospital of Joint Logistics Support Force of Chinese People’s Liberation Army, Lanzhou, Gansu Province, China; b The Clinical Medical College of Ningxia Medical University, Yinchuan, Ningxia Hui Autonomous Region, China; c The First Clinical Medical College of Gansu University of Chinese Medicine, Lanzhou, Gansu Province, China; d Department of Orthopedics, The First Clinical Medical College of Lanzhou University, Lanzhou, Gansu Province, China; e Orthopedic Center, The 940^th^ Hospital of Joint Logistics Support Force of Chinese People’s Liberation Army, Lanzhou, Gansu Province, China.

**Keywords:** case report, multiple organ failure, necrotizing fasciitis, skin necrosis, treatment

## Abstract

Necrotizing fasciitis (NF) is a rare, severe soft tissue infection, characterized by rapid and extensive necrosis of the skin, subcutaneous tissue, and superficial and deep fascia. It is frequently misdiagnosed as other infectious diseases, leading to inappropriate treatment and potentially serious consequences. It may be complicated by septic shock and multiple organ failure with a fatal outcome.

**Patient concerns:**

A 73-year-old woman presented with continuous itching, skin lesions, pain, and swelling of the outer side of her left leg. The patient was diagnosed with septic shock and multiorgan failure caused by left leg NF.

**Diagnosis:**

Septic shock and multiorgan failure caused by left leg NF.

**Interventions:**

Two surgeries were performed on the patient’s leg, which effectively treated her septic shock and multiple organ dysfunction.

**Outcomes:**

The patient was followed up three times after her discharge. She had a good recovery, was generally well with no significant sequelae, and returned to her regular life.

**Conclusion:**

NF is an acute severe illness with high mortality. It is easily misdiagnosed, leading to delayed or erroneous treatment and serious (or potentially fatal) outcomes. Rapid and accurate diagnosis of NF is essential for patient recovery. In difficult cases, multidisciplinary consultations may be helpful. The management of NF includes early and thorough surgical debridement, antibiotics, and symptomatic treatment.

## 1. Introduction

Necrotizing fasciitis (NF) is a rare, life-threatening soft tissue infection of the deep and superficial fascia; it causes necrosis of the skin, subcutaneous tissue, and fascia, but usually spares the muscles. NF patients are at a risk of developing sepsis, septic shock, and multiple organ failure, with a high mortality rate (6–76%) and potentially fatal outcomes.^[1–3]^ NF may be misdiagnosed as a common skin and soft tissue infection, thereby leading to erroneous treatment. Here we report a case of NF with delayed diagnosis and treatment due to the inexperience of the attending physician, and subsequent correct diagnosis and good recovery after multidisciplinary consultations.

## 2. Case presentation

A 73-year-old woman developed sustained itching on the left leg 4 days before presentation. Scratching of the skin led to skin damage, redness, swelling, and pain. Before presentation to the hospital, the patient applied an unknown ointment, which did not improve her symptoms. The left leg pain and swelling gradually increased; however, the involved area did not increase. Three days later, the patient developed severe diarrhea and passed almost 20 stools per day. The stools were yellowish green and watery. The patient also developed fecal incontinence. At presentation to the emergency department, she was in shock and had a blood pressure of 42/30 mm Hg. She was immediately resuscitated with rehydration and norepinephrine infusion to increase the blood pressure. An urgent abdominal ultrasound showed a slightly enlarged fatty liver and spleen. Color Doppler echocardiography showed an enlarged left atrium, left ventricular ejection fraction of 52%, and left ventricular short axis shortening of 21%. Computed tomography (CT) of the left leg showed soft tissue swelling in the middle and lower segments, blurred subcutaneous fat space with grid changes, significantly thickened deep fascia, and normal morphology and density of the left tibia and fibula. The CT findings were suggestive of infection. The laboratory findings revealed a C-reactive protein of 153 mg/L, procalcitonin (PCT) of > 25.0 ng/mL, and N-terminal B-type natriuretic peptide precursor of 11, 349 pg/mL. The patient was evaluated by the critical care physicians and admitted with diagnoses of septic shock and multiple organ failure.

The patient had a history of hypertension for more than 20 years, and her highest blood pressure was recorded at 180/112 mm Hg. She was regularly taking nitrendipine and telmisartan for her hypertension, and her blood pressure was usually at 130/80 mm Hg. She underwent hysterectomy for hysteromyoma almost 20 years ago; however, additional information was not available. There was no history of food or drug allergy, and no relevant personal or family history.

**Figure 1.**
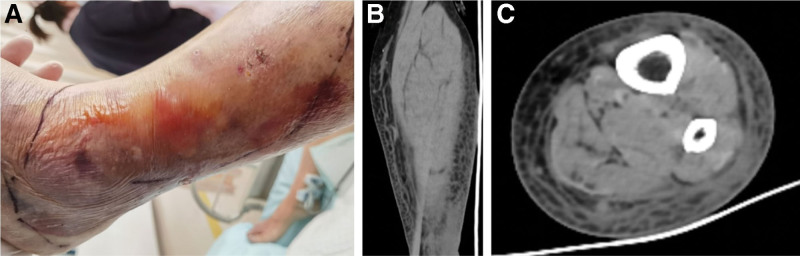
(A) At the time of intensive care unit admission, the patient’s lower left leg exhibited redness, swelling, ulcers, blisters, and pus spots on the lateral surface. (B, C) CT of the left leg showed soft tissue swelling in the middle and lower segments, blurred subcutaneous fat space, grid changes, and significantly thickened deep fascia. CT = computed tomography.

At the time of intensive care unit admission, the patient’s temperature was 38.4°C; her pulse rate was 141 beats/minute, respiratory rate was 28/minute, blood pressure was 68/38 mm Hg, and visual analog scale score for pain was 5. The patient was administered high-flow humidified oxygen (O_2_ concentration of 50%, flow of 6 L/minute), after which her oxygen saturation was 90%. She had intact consciousness and a poor mental state. She was able to provide brief responses to questions. Her bilateral pupils were round and equal in size, with normal light reflex. Auscultation revealed dry and wet rales in both lungs. The heart rate and sounds were irregular, and the heart rate was higher than the pulse rate. The abdomen was flat and soft, with no tenderness or rebound pain. She had hyperactive bowel sounds audible almost 8 times/minute. The extremities were cold and the lateral skin of the lower part of the left leg was significantly red, swollen, and warm. Her leg exhibited multiple ulcers and blisters, with pus exudates (Fig. 1A). She had leg tenderness, normal joint movements, and negative Babinski sign.

Abdominal ultrasound performed at admission showed slightly enlarged fatty liver and spleen. Color Doppler echocardiography showed left atrial enlargement, left ventricular ejection fraction of 52%, and left ventricular short axis shortening of 21%. CT of the left leg showed soft tissue swelling of the middle and lower segments, subcutaneous fat space blurring and grid changes, significantly thickened deep fascia, and almost normal morphology and density of the left tibia and fibula. The imaging findings were suggestive of infection (Fig. 1B and C).

Laboratory examination revealed a white blood cell count of 19.77 × 10^9^/L, hemoglobin level of 103 g/L, neutrophil proportion of 90.5%, platelet count of 53 × 10^9^/L, activated partial thromboplastin time of 39.5 seconds, prothrombin time (PT) of 20.3 seconds, international normalized ratio of 1.75, PT activity of 39.9%, PT ratio of 1.72, thromboplastin time of 17.1 seconds, fibrinogen level of 4.60 g/L, fibrinogen degradation product level of 53.80 μg/mL, D-dimer level of 16.24 mg/L, procalcitonin level of > 100 ng/mL, C-reactive protein level of 153 mg/L, interleukin-6 level of > 5000.0 pg/mL, pH of 7.29, lactic acid level of 6.3 mmol/L, partial pressure of O_2_ (PO_2_) of 32 mm Hg, partial pressure of CO_2_ of 48 mm Hg, oxygen saturation of 61%, alkali residue of–3.7 mmol/L, oxygenation index (PO_2_/FiO_2_) of 64 mm Hg, potassium level of 3.03 mmol/L, sodium level of 137.5 mmol/L, glucose level of 21.1 mmol/L, N-terminal B-type natriuretic peptide precursor level of 11, 349 pg/mL, myoglobin level of 584.2 μg/L, troponin level of 0.065 μg/L, lactate dehydrogenase level of 326 IU/L, creatine kinase level of 309 IU/L, aspartate aminotransferase level of 101 IU/L, alanine aminotransferase level of 79 IU/L, total protein level of 56.2 g/L, albumin level of 32.1 g/L, albumin/globulin ratio of 1.3, total bilirubin level of 46.07 μmol/L, direct bilirubin level of 28.52 μmol/L, indirect bilirubin level of 17.55 μmol/L, cholic acid level of 14.2 μg/mL, alkaline phosphatase level of 120 IU/L, creatinine level of 347.0 μmol/L, urea level of 15.50 mmol/L, B2-MG of 11.62 mg/L, prealbumin level of 87 mg/L, and estimated glomerular filtration rate of 10.69 mL/minute.

**Figure 2.**
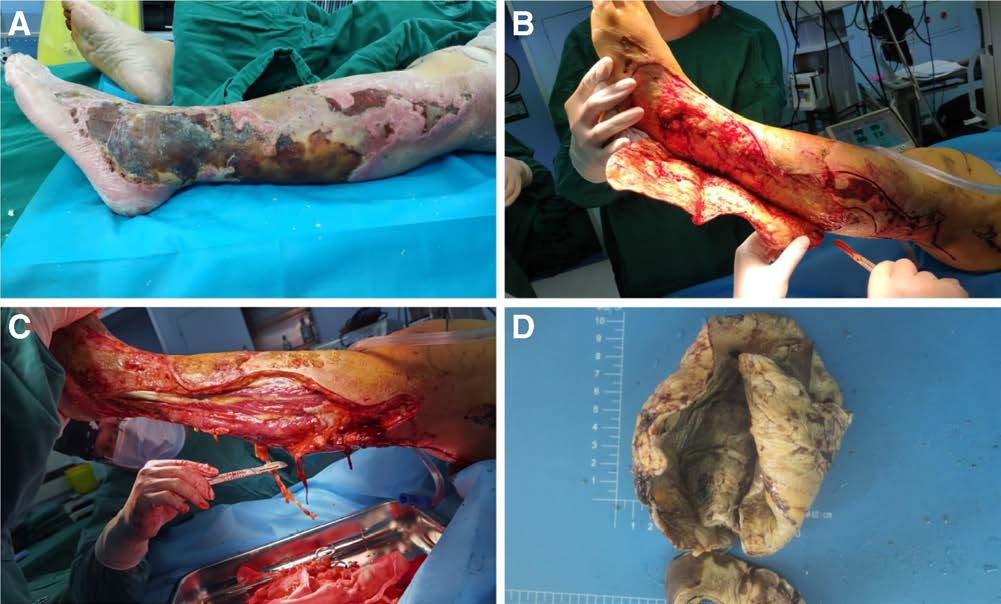
(A) Almost 10 h after admission, the skin over the patient’s left leg gradually changed from red to purple and black, with large patches of flower-like appearance. The discoloration spread to the feet, and there was a foul-smelling pus exudate. (B, C) During the first debridement, we performed “carpet-like” debridement of the necrotic skin of the patient’s left leg, lateral ankle, and dorsum of the foot, removed the necrotic skin over the anterolateral and posterior sides of the leg below the middle of the left leg and the underlying inactivated fat and fascia. We performed appropriate extensive debridement of the lateral ankle and dorsum of the foot. (D) An adequate sample of necrotic tissue was sent to the Department of Pathology for examination.

After admission to the intensive care unit, the patient’s vital signs were monitored, followed by endotracheal intubation and ventilator-assisted breathing. She was treated with high-flow oxygen, analgesia, sedation, fluids, and blood products for resuscitation, norepinephrine and epinephrine to increase the blood pressure, a traditional Chinese medicine to restore the Yang and reverse the shock, meropenem (0.5 g, ivgtt, q 6h) and vancomycin (0.5 g, ivgtt, q 12h) to treat the infection, amiodarone to correct the heart rate, bromhexine hydrochloride and sputum aspiration to improve expectoration, lansoprazole to inhibit gastric acid secretion and prevent stress ulcers, low-molecular-weight heparin for anticoagulation, continuous renal replacement therapy, and supportive management to correct the electrolyte disorders. The patient’s shock improved and the number of stool episodes decreased. After 10 hours of admission, the color of patient’s left leg gradually changed from red to purple and black, and foul-smelling pus exudates. The discoloration gradually spread to the feet (Fig. 2A). There were reduced sensations on the skin and the symptoms of septic shock worsened. She was resuscitated again. The blood, sputum, and pus bacterial culture and identification tests were repeated; however, the results were expected after 3 days. The attending physician diagnosed the patient with a common skin infection. The patient could not be diagnosed despite discussion with the other doctors of the department. Therefore, multidisciplinary consultations were sought from the departments of infectious diseases, gastroenterology, general surgery, dermatology, burns and plastic surgery, and trauma and orthopedics on the fourth day of admission. The doctors from the departments of orthopedics and general surgery diagnosed the skin necrosis as NF. The diagnosis was confirmed using the Laboratory Risk Indicator for Necrotizing Fasciitis (LRINEC) score. The patient had a LRINEC score of 10 (total score: 13), suggesting the diagnosis of NF and need of debridement. Considering the rapid spread of the necrotic area and presence of septic shock, the treatment was initiated before the results of bacterial culture and identification were available. On the same day, the culture of pus obtained from the left leg showed *Streptococcus pyogenes*, the sputum culture showed *Acinetobacter baumannii*, and the blood culture showed no growth. In accordance with the drug sensitivity results, we replaced the patient’s antibiotics with cefoperazone sodium, sulbactam sodium (3 g, ivgtt, q 8h), and tigecycline (100 mg, ivgtt, q 12h). We informed the patient’s family members regarding the patient condition and treatment plan, and suggested urgent surgical intervention (left leg debridement and wound closure with negative pressure drainage) to remove the source of pathogenic bacteria and toxins, followed by additional operations (left leg debridement and wound skin grafting), once the patient improves. The family members agreed with our diagnosis and treatment plan.

**Figure 3.**
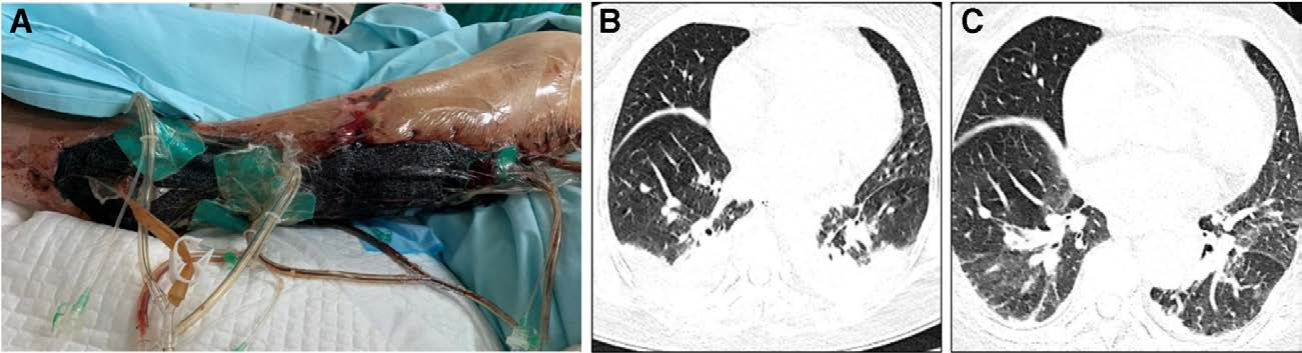
(A) After debridement, we closed the wound with VSD and inserted a drain into the wound. (B) On the second postoperative day, the patient’s chest CT showed patchy shadows in the upper and lower lobes of both lungs and bilateral pleural effusion. (C) On the tenth postoperative day, the CT showed significantly reduced exudate shadows in both lungs and pleural effusion. CT = computed tomography, VSD = vacuum sealing drainage.

After active resuscitation and treatment, patient’s shock, diarrhea, and general condition gradually improved. After evaluation by the anesthesiologist, the patient underwent debridement of the left lower limb and wound closure with negative pressure drainage on the tenth day after admission. The intraoperative “finger test” on the patient’s left leg was positive, and the index finger was able to touch the fascia with little resistance and only minor bleeding. In addition, the finger could easily separate the subcutaneous tissue from the fascia. The necrotic skin over the left calf, lateral ankle, and dorsum of the foot was debrided in a “rolled carpet” manner. The necrotic skin over the anterolateral and posterior surfaces of the middle and lower sections of the left calf, as well as the underlying inactivated fat and fascia, were removed. Then, extensive debridement was performed for the skin overlying the lateral ankle and dorsum of the foot (Fig. 2B and C). Once the skin was debrided, yellow pus flowed out. A sample of the necrotic tissue was sent for pathological analysis (Fig. 2D). The wound was repeatedly washed with a large quantity of normal saline, 3% hydrogen peroxide, and iodophor solution. The wound was closed and vacuum sealing drainage (VSD) was applied (Fig. 3A). The patient’s preoperative laboratory test results showed a red blood cell count of 2.51 × 10^12^/L, hemoglobin level of 70 g/L, and hematocrit of 24.6%. In addition, there was significant intraoperative bleeding. Therefore, the patient was administered 2.5 U of suspended red blood cells and 496 mL frozen plasma during the operation. The operation was successful and the patient was returned safely to the ward.

We repeated the imaging and laboratory examinations postoperatively. On the second postoperative day, the patient’s dyspnea worsened and auscultation revealed wet rales in both lungs. Chest CT showed patchy shadows in the upper and lower lobes of both lungs, and bilateral pleural effusion (Fig. 3B). The patient was treated with auxiliary breathing techniques, sputum aspiration, thoracic puncture and drainage, and antibiotics (cefoperazone sodium and sulbactam sodium; 3 g, q 8h). On the tenth postoperative day, chest CT showed significant improvements in bilateral pulmonary exudates and pleural effusion (Fig. 3C). The patient was weaned from the ventilator and maintained on sequential high-flow oxygen via a mask. The patient had stable breathing and circulation. However, the patient required continuous renal replacement therapy due to renal insufficiency.

**Figure 4.**
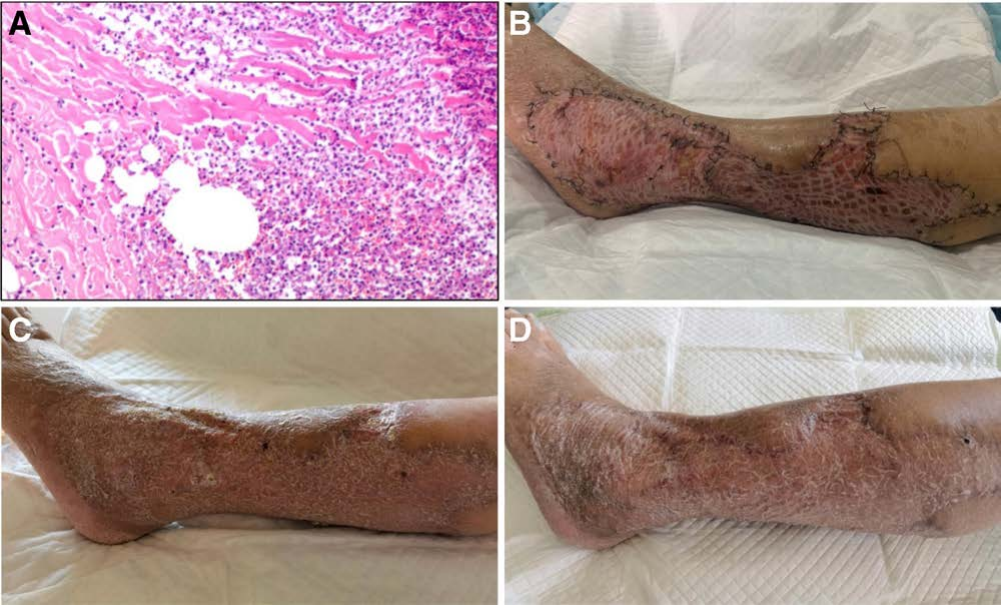
(A) On the fifth postoperative day, the pathological analysis showed fibrous tissue proliferation, extensive necrosis, and infiltration of lymphocytes and neutrophils. (B) On the tenth day after the second operation, the transplanted skin over the left leg exhibited good growth and no signs of infection or necrosis. (C) At the first follow-up (3 months after discharge), the transplanted skin exhibited good growth, wound healing, and scabs over the surface. (D) At the second follow-up (6 months after discharge), the skin and wound over the left leg exhibited wound healing and reduced scab area.

On the fifth postoperative day, the pathological analysis of tissue obtained from the left leg showed fibrous tissue proliferation, large areas of necrosis, and infiltration of lymphocytes and neutrophils (Fig. 4A). Postoperatively, the patient was treated with antibiotics, blood transfusion, gastric ulcer prophylaxis, liver protective agents, anticoagulants, nutritional support, and other supportive treatments. Subsequently, the patient’s mental state and appetite improved significantly, and her breathing and circulation stabilized. The VSD over the left leg was maintained for 1 week and no leakage was observed. The orthopedic surgeon reevaluated the patient and suggested that the surgical wound was significantly improved and recommended additional debridement and skin transplantation, depending on the wound condition.

The patient’s condition was reassessed and repeat debridement of the left leg and skin transplantation were performed. After removing the VSD, we repeated the debridement of the left leg wound, ensured hemostasis, and washed and covered the wound with sterile wet gauze. Then, we obtained two medium thickness skin grafts (20 × 10 cm) applied them over the wound. Dressing was applied over the graft, and the surgical area was wrapped with sterile dressing. On the tenth day after the second operation, we removed the dressing from the skin graft over the left leg. The transplanted skin showed good growth and did not exhibit any signs of infection or necrosis (Fig. 4B). After the surgical and medical treatment, the patient’s mental and general conditions improved significantly, and she was able to sit up and eat by herself. She had improved hemodynamic parameters, urine volume, and laboratory test results. After the renal function improved, continuous renal replacement therapy was stopped. Then, we transferred the patient to the orthopedic center for the functional exercises of the left leg. During the patient’s hospitalization, we played her favorite music on the mobile phone and chatted with her. In addition, she was allowed to meet her family members. As a result, the patient’s mental and psychological state was improved, and she cooperated with the treatment, thereby leading to a good outcome.

The patient was followed-up at 3, 6, and 12 months after discharge. At the 3-month follow-up, the patient’s mental state, appetite, and urination were normal, while the respiratory, circulatory, and urinary examinations were not significantly abnormal. The transplanted skin showed good growth, almost complete healing of the wound, and extensive scabbing (Fig. 4C). The leg had a muscle strength of grade IV and low muscle tone. She was able to walk with the help of crutches. Her laboratory test results were almost normal. At the 6-month follow-up, the left leg skin and wound were healed, and the scab was reduced in size (Fig. 4D). The patient was able to walk without the support of crutches, but limped while walking. The 12-month follow-up was conducted over telephone. The patient reported that she had a good physical condition and walked with a slight limp; however, the limp did not significantly affect her activities of daily living. She was satisfied with her recovery, but did not provide us with photographs of the surgical site.

## 3. Discussion

NF is a relatively rare bacterial infection and has a rapid onset and progression. It causes significant destruction to the skin and soft tissue. Most causes are caused by bacterial infection, which destroys the skin integrity. The main pathogenic bacteria that cause NF are *Staphylococcus aureus*, *Streptococcus pyogenes*, *Klebsiella pneumoniae*, *Vibrio vulnificus*, and *Aeromonas hydrophila*.^[4]^ Fungi can also cause NF.^[5]^ NF can be divided into types I (caused by mixed bacteria, including anaerobic bacteria, gram-positive cocci, and gram-negative bacilli), II (single strain), III (single gram-negative strain), and IV (fungal infection); of these, types I and II are the most common and account for more than 90% of cases.^[6,7]^ Our patient had NF type II. NF can occur at any age, but it is particularly common in elderly men and very rare in children.^[5,8]^ It tends to affect the limbs, trunk, perineum, and other body parts^[9–12]^ (Table 1). The risk factors of NF include old age, smoking, alcohol use, obesity, diabetes, peripheral vascular disease, liver disease, tumors, and prolonged high-dose steroid use, of which diabetes is the most common risk factor.^[13]^ Studies have found that almost 53.3% of NF patients have diabetes.^[14,15]^ Our patient did not have diabetes or history of prolonged steroid use. COVID-19 pneumonia may also cause NF; however, the underlying mechanism is not clear.^[16]^

**Table 1:** Studies of necrotizing fasciitis worldwide.

Ref.	Age (y), Sex	History of diabetes	Predisposing factors	Location of disease	Main clinical manifestations	Pathogenic species	Therapeutic method	Outcome
Bottger *et al*^[1]^	71 (females), 65 (females), 74 (males), 38 (males)	One patient had a history of diabetes (4 patients in total)	Chronic apical periodontitis	Neck and chest	Pain, swelling, black blisters, livid erythema, and poor general condition	*Prevotella intermedia*, alpha hemolytic streptococci, *Candida albicans*.	Tooth removal, skin graft, tracheostomy, and antibiotics (meropenem, imipenem,and metronidazole)	3 patients recovered, 1 died
Petreanu *et al*^[2]^	43, male	Not reported	Left pulmonary tuberculosis	Walls of the chest	Severe dyspnea and cough with mucopurulent sputum	*Pseudomonas aeruginosa*, methicillin-resistant Staphylococcus,	Surgical debridement, left lung resection,and antibiotic use (not reported)	Recovery
William *et al*^[5]^	Premature infants (11 days), male	Not reported	Poor feeding	Neck and maxilla	Shortness of breath, neck and maxillary erythema, and skin necrosis	cutaneous mucormycosis	Surgical debridement, antibiotic use (augmentin, meropenem, vancomycin, colistin, and amphotericin B)	Recovery
Xu *et al*^[9]^	61, male	Yes	Using high doses of glucocorticoids	Right lower limb	Redness over the back of the foot, local tenderness, and higher skin temperatures	*Staphylococcus aureus*	Surgical debridement, Antibiotic use (cefoperazone sodium/sulbactam sodium/caspofungin,linezolid)	Recovery
Joomun *et al*^[10]^	56, female	Yes	Traffic collision	Left lower extremity	Severe pain and swelling, septic shock, and multiple organ failure	Not reported	Surgical debridement, skin transplantation,and antibiotic use (not reported)	Death
Aziret *et al*^[11]^	36, female	No	C-section	Abdomen	Chills, fever, surgical site infection, and acute abdomen	Not reported	Surgical debridement, antibiotic use (ceftriaxone, metronidazole, meropenem,and teicoplaninum), high-dose systemic corticosteroids	Recovery
Tao *et al*^[12]^	50, male	No	Not reported	Perianeum and scrotum	Anal pain and discomfort, and perianal abscess	Not reported	Surgical debridement,antibiotic use (fourth-generation cephalosporin and metronidazole)	Recovery
Zhan g *et al*^[13]^	56, female	Yes	Diabetes	Right foot and thigh	Skin swelling, ulcer,and tissue necrosis of the right foot and thigh	Not reported	Surgical debridement, amputation,antibiotic use (vancomycin and imipenem)	Recovery
Elashry *et al*^[16]^	52, male	No	COVID-19	Abdomen	High-grade fever, Chills, abdominal distension and pain,cutaneous erythematous patch	Klebsiella, *Escherichia coli*, and mixed anaerobic species	Surgical debridement and antibiotic use (not reported)	Recovery
Sammoni *et al*^[28]^	16, female	No	Minor trauma on her left palm	Back, abdomen, and armpits	Pain, tenderness, and erythema	GAS	Surgical debridement, antibiotic use (clindamycin, vancomycin, ceftazidime, fluconazole, piperacillin/tazobactam,and tigecycline)	Recovery

COVID-19 = Coronavirus disease 2019; GAS = Group A streptococcus.

Wang *et al*^[17]^ proposed three clinical stages of NF. In the early stage, skin at the infection site is swollen, warm, and severely painful; the pain is out of proportion to the other signs. In the intermediate stage, the affected skin develops severe ischemia, thrombosis of blood vessels supplying the fascia, and blisters with serous fluid. In the late stage, the infected skin becomes necrotic, gray, and dark. In addition, blood vessels supplying the fascia are blocked, there is sensory loss (particularly for pain and touch sensations), and there is gas production from the tissues, which produces a “twisting sound” on palpation. Therefore, our patient was at the late stage of NF when she underwent surgical debridement.

Because of the occult onset and rapid progression of NF, delayed diagnosis or erroneous diagnosis or treatment may occur, leading to a high mortality rate of NF. Therefore, early diagnosis is essential to achieve a good outcome. The gold standard diagnostic tests for NF include surgical exploration and histopathological and microbiological analyses. Intraoperatively, NF exhibits extensive necrosis of the fascia. The fascia is easily separated from the surrounding tissues with no significant bleeding, and there is a large quantity of foul-smelling purulent discharge. However, NF is difficult to diagnosis during surgical exploration before the debridement. The “finger test” was introduced by Andreasen^[18]^ and is similar for the preoperative diagnosis of NF during surgical exploration. It is a common method used for the diagnosis of NF. For this method, local anesthesia is administered, a 2 cm wide incision is made from the infected area down to the deep fascia, and the fascia is palpated with the index finger. The test is considered positive (i.e., NF is present) if there is no significant bleeding and little resistance, the subcutaneous tissue is easily separated from the fascia, and there is necrosis of the fascia and large quantity of pus discharge. Urgent surgery is required for patients with rapid progression and severe disease, and NF can be confirmed intraoperatively based on the appearance of the soft tissue. Tissue samples should be sent for pathological and microbiological analyses, including tissue culture and bacteria identification.

The LRINEC score was proposed by Wong *et al*^[19]^ in 2004 and is commonly used for the diagnosis of NF. LRINEC has a total of 13 points, with scores of ≤ 5, 6–7, and ≥ 8 correlated with NF incidences of < 50%, 50‐75%, and > 75%, respectively. Large-scale studies have verified the usefulness of LRINEC for the early diagnosis of NF.^[20]^ Our patient had a LRINEC score of 10 before the surgical debridement, which increased the reliability of our preoperative diagnosis. Magnetic resonance imaging (MRI) is the preferred imaging modality and provides high-resolution soft tissue images. Fat-suppressed T2-weighted imaging reveals thickening of the deep fascia, effusion, and high signals; it has a sensitivity of 100% and specificity of 86%.^[21]^ MRI is the most effective method for differentiating between NF and non-NF infections. However, performing an MRI is time-consuming. MRI may not be suitable for patients who are critically ill or require instruments and equipment that are not compatible with MRI. Such patients may benefit from CT, which may show thickened fascia due to reactive hyperemia, subcutaneous effusion, gas accumulation, fat grid reinforcement, and muscle asymmetric reinforcement.^[22]^ X-ray is rarely used for the diagnosis of NF. Ultrasound demonstrates thickened subcutaneous tissue and fascia, localized effusion around the fascia, scattered strong echo in subcutaneous tissue, and cobblestone-like changes.^[23]^ No single imaging examination can confirm the diagnosis of NF preoperatively, and the diagnosis should be based on the clinical manifestations and several auxiliary investigations. In difficult cases, multidisciplinary consultations for the diagnosis and treatment may lead to a good outcome.

Surgical debridement and negative pressure VSD are the most commonly used and effective methods for the treatment of NF; they remove the source of pathogenic bacteria and toxins, and prevent further necrosis of tissues. Early debridement is associated with improved outcomes, and surgical debridement after 24 hours of symptom onset is associated with a 9-fold higher mortality rate compared to debridement within 24 hours.^[24]^ During debridement, the necrotic skin, subcutaneous tissue, and fascia, along with the dead corners, must be completely removed. To ensure the effectiveness of debridement and reduce the number of debridement procedures needed, it should be performed during wound exploration. Tissues that are not completely necrotic but have poor blood supply and vitality must also be removed, and the debridement area may be expanded if necessary. The debrided wound should be washed several times with a large quantity of normal saline, hydrogen peroxide, and iodophor solution. Adequate sample area should be obtained from the necrotic tissue and pus for pathological examination and bacterial culture and identification. After debridement, VSD is used to cover and seal the wound to improve healing. Continuous negative pressure to the wound effectively removes the exudate, reduces the absorption of toxins and surrounding edema, stimulates local blood circulation, and promotes the growth of granulation tissue and wound healing.^[25–27]^ However, VSD cannot replace surgical debridement. Sammoni *et al*^[28]^ reported a closed lavage technique for the treatment of back NF, which has a similar principle to VSD and leads to good results. Once the wound matures, skin transplantation should be performed to close the wound.

Antibiotic use is essential during NF treatment. Because NF is usually caused by multiple bacterial strains, broad-spectrum antibiotics that are effective against Gram-positive and Gram-negative bacteria (e.g., vancomycin and meropenem) should be used empirically. When anaerobic or fungal infection is suspected, metronidazole or voriconazole should be added to the treatment. When the pathogen is identified, the antibiotics should be adjusted accordingly. Hyperbaric oxygen has been used for the treatment of NF after debridement because it eliminates anaerobes, increases leukocyte activity, inhibits the production of inflammatory mediators by increasing the concentration of blood oxygen, and provides additional oxygen to the hypoxic tissues surrounding the necrosis.^[29,30]^ However, some studies have reported uncertain effectiveness of hyperbaric oxygen for the treatment of NF. Therefore, additional clinical studies are needed to confirm the effect of hyperbaric oxygen. Most patients with NF are seriously ill and require several medical equipment required to maintain hemodynamic stability, which makes it difficult to administer hyperbaric oxygen treatment.

NF progresses rapidly, and most patients with NF develop sepsis, septic shock, and multiple organ failure. Several medical equipment and drugs are required to maintain the hemodynamic stability, breathing, circulation, and renal function. The symptomatic treatment includes sedation, analgesia, blood transfusion, blood glucose control, and correction of water, electrolyte, and acid-base imbalance. NF patients often have a prolonged disease course, increased basal metabolic rate, and high energy consumption; therefore, the nutritional intake of patients should be increased accordingly. The mental and psychological states of patients should also be evaluated. Many NF patients develop irritability, anxiety, and depression. They can be supported by chatting with them, playing their favorite music on the mobile phones, and letting their family members meet the patients. If the mental and psychological state of patients does not improve, drugs can be administered.

## 4. Conclusion

NF is an acute severe illness with high mortality, and is easily misdiagnosed as a common skin and soft tissue infection, which delays the treatment and leads to worsened patient condition and even death. Rapid and accurate diagnosis of NF is essential for a good outcome. In difficult cases, multidisciplinary consultations for the diagnosis and treatment may be helpful. Early and thorough surgical debridement is the main treatment of NF. The use of antibiotics and symptomatic treatment are also essential.

## Author contributions

**Funding acquisition:** Xiao-Hui Yu.

**Investigation:** Zhao-Jun Liang, Bao-Yin Zhao.

**Methodology:** Zhao-Jun Liang, Bao-Yin Zhao, Tao Zhang, Hua Liu.

**Resources:** Li-Xia Zhang, Xue-Wen Shi, Tao Zhang, Hua Liu.

**Visualization:** Xue-Wen Shi.

**Writing – original draft:** Li-Xia Zhang.

**Writing – review & editing:** Xiao-Hui Yu.
